# Planting conditions can enhance the bioactivity of mulberry by affecting its composition

**DOI:** 10.3389/fpls.2023.1133062

**Published:** 2023-03-07

**Authors:** Huixin Bai, Shanfeng Jiang, Jincai Liu, Ye Tian, Xiaohui Zheng, Siwang Wang, Yanhua Xie, Yao Li, Pu Jia

**Affiliations:** ^1^ Department of Life Science and Medicine, Northwest University, Xi’an, China; ^2^ School of Life Sciences, Northwestern Polytechnical University, Xi’an, China; ^3^ School of Pharmacy, Shaanxi University of Chinese Medicine, Xianyang, China

**Keywords:** mulberry branch, planting areas, planting conditions, bioactivities, partial least square regression, multiple factor analysis

## Abstract

Mulberry (*Morus alba* L.) has a special significance in the history of agriculture and economic plant cultivation. Mulberry has strong environmental adaptability, a wide planting range, and abundant output. It is not only an important resource for silkworm breeding but also a raw ingredient for various foods and has great potential for the development of biological resources. The bioactivities of mulberry in different planting areas are not the same, which is an obstacle to the development of mulberry. This study collected information on the planting conditions of mulberry branches in 12 planting areas, such as altitude, temperature difference, and precipitation. A comparison of the levels of 12 constituents of mulberry branches from mulberry grown in different planting areas was then made. An *in vitro* model was used to study the bioactivities of mulberry branches in the 12 planting areas, and mathematical analysis was used to explain the possible reasons for the differences in the composition and bioactivities of mulberry branches in different planting areas. After studying mulberry samples from 12 planting areas in China, it was found that a small temperature difference could affect the antiapoptotic effect of mulberry branch on microvascular endothelial cells by changing the levels and proportions of rutin, hyperoside, and morusin. Adequate irrigation can promote the antioxidation of the mulberry branch on microvascular endothelial cells by changing the levels and proportions of scopoletin and quercitrin. The results of the analysis of planting conditions and the levels of active constituents and their correlation with bioactivities support the improvement of mulberry planting conditions and have great significance in the rational development of mulberry resources. This is the first time that a mathematical analysis method was used to analyze the effects of planting conditions on mulberry biological activity.

## Introduction

Mulberry (*Morus alba* L.) has a special significance in the agricultural and economic plant cultivation history of China. It is not only an important resource for silkworm breeding, but also a source of various foods and teas, and has great application potential in biological resources (Chao et al., 2021). Owing to its great adaptability to different climates, mulberry has unique advantages as a natural resource and is widely distributed across China. From Heilongjiang province in the north to Guangxi and Guangdong in the south, it spans the entire geographic latitude of China. Thus, the significant differences in planting conditions in these areas, such as altitude, precipitation, and temperature difference, have become an important factor affecting mulberry development. In addition, because mulberry trees have strong germination (Hashemi and Tabibian, 2018) and rapid regeneration after pruning, a large amount of pruning must be carried out every year to facilitate the normal growth of the trees, which produces a large number of mulberry branches and leaves. However, only the mulberry leaves and fruits, and not the branches, are fully developed and utilized. The mulberry branch is crushed to make fertilizer or simply burned (Yin et al., 2017) which is a waste of mulberry plant resources.

Mulberry branches are rich in alkaloids, flavonoids, polysaccharides, etc. The active constituents of the mulberry branch, such as chlorogenic acid, mulberroside A, resveratrol, and 1-deoxynojirimycin (1-DNJ), can prevent diabetes and related complications and protect pancreatic cells from oxidative damage (Han et al., 2020; Hou et al., 2020). For example, chlorogenic acid inhibits inflammation and fat deposition in the liver by reducing energy intake and food efficiency, and it also increases the diversity of the gut microbiota, thereby improving overall metabolism. Long-term consumption of chlorogenic acid is beneficial for cardiocerebral vessels, the liver, and the metabolism (Bhandarkar et al., 2019). Morusin is a kind of flavonoid derived from mulberry that has a strong antioxidant capacity and can repress oxidative stress, thus protecting the integrity of pancreatic β-cells and reducing cell death. Morusin can also improve hyperglycemia and lipid homeostasis in type I diabetic mice induced by streptozotocin (Choi et al., 2020). Cortex Mori water extract, containing mulberroside A, reduces blood glucose, thus alleviating liver and kidney damage caused by hyperglycemia, and ameliorates diabetic endotoxemia (Xu et al., 2021). Resveratrol has been used in various anti-hyperglycemia studies because of its physiological effects of lowering levels of blood sugar, improving insulin sensitivity, and protecting pancreatic β cells (Öztürk et al., 2017; Zhu et al., 2017; Huang et al., 2020). Studies have found that scopoletin can inhibit postprandial blood glucose levels and that the mechanism of action is related to the inhibition of α-glucosidase and α-amylase activity (Jang et al., 2018). 1-deoxynojirimycin (1-DNJ) is a potent α-glucosidase inhibitor, which can suppress the increase of blood sugar before and after meals without serious side effects (Thaipitakwong et al., 2020). It can also inhibit hypercholesterolemia induced by a high-fat diet (HFD) and regulate the gut microbiota (Li et al., 2019).

The mulberry branch and mulberry leaf are similar in their compositional basis and bioactivities (Bai et al., 2023), and the abundant yield of mulberry branches has obvious advantages for exploitation. However, there is no research that reveals the impact of the planting conditions of mulberry planting areas on the levels of the constituents of the mulberry branch. The lack of verification of the functional similarity of mulberry branches in different planting areas hinders the development and research of mulberry resources. Therefore, in this study, by determining fingerprint morphology and main constituent levels, the compositional similarity of mulberry branches from 12 planting areas in China was compared using the mulberry leaf as a control. A hyperglycemia and hyperlipidemia model (Bai et al., 2023) was used to study the effects of different planting areas on the mulberry branch on apoptosis and antioxidant damage *in vitro*. Partial least square regression (PLSR) and multiple factor analysis (MFA) were used to analyze the effects of planting condition factors on the constituents and bioactivities of mulberry branch extracts. This research provides a theoretical basis for improving mulberry planting conditions and rationally developing mulberry resources. This is the first time that the mathematical analysis method has been used to analyze the effects of planting conditions on the constituents and bioactivities of the mulberry branch.

## Materials and methods

### Instruments and reagents

The mulberry branch and leaf were identified as belonging to dry branches and leaves of *Morus alba* L. by Ling-bian Sun, the chief pharmacist of the Air Force Medical University (Xi’an, China). The mulberry leaf was purchased from Shiquan County, Shaanxi, China. Mulberry branches were purchased from different planting areas, as shown in **Table 1**.

**Table 1:** Planting conditions of mulberry from different planting areas.

Planting areas	Latitude and longitude	Altitude (m)	Annual temperature difference (°C)	Total annual precipitation (mm)	Rain concentrated (month)	Month before harvest	
						Temperature difference (°C)	Precipitation (mm)
S1 (20180401)	31°42′N–39°35′N, 105°29′E–111°15′E	396.9	28	565	7–9	29	18
S2 (20180502)	43°25′N–53°33′N, 121°11′E–135°05′E	171.7	42	554	6–8	28	72
S3 (20180520)	38°43′N–43°26′N, 118°53′E–125°46′E	41.6	37	662	6–8	25	76
S4 (20180530)	36°01′N–42°37′N, 113°04′E–119°53′E	80.5	30	540	7–9	31	27
S5 (20180408)	31°42′N–39°35′N, 105°29′E–111°15′E	396.9	28	565	7–9	29	18
S6 (20180416)	31°23′N–36°22′N, 110°21′E–116°39′E	110.4	28	617	7–9	31	12
S7 (20180415)	30°45′N–35°20′N, 116°18′E–121°57′E	8.9	26	1275	6–8	25	91
S8 (20180417)	29°41′N–34°38′N, 114°54′E–119°37′E	29.8	26	1148	6–8	28	93
S9 (20180310)	27°02′N–31°11′N, 118°01′E–123°10′E	41.7	25	1639	6–8	28	140
S10 (20180408)	26°03′N–34°19′N, 97°21′E–108°31′E	505.9	20	980	7–9	20	21
S11 (20180413)	21°08′N–29°15′N, 97°31′E–106°11′E	1891.4	12	933	6–9	20	25
S12 (20180401)	20°54′N–26°23′N, 104°29′E–112°04′E	72.7	16	1357	6–8	21	24
S13 (20180420)	20°09′N–25°31′N, 109°45′E–117°20′E	6.6	15	2084	5–7	28	130

S1, Mulberry leaf; S2, Heilongjiang; S3, Liaoning; S4, Hebei; S5, Shanxi; S6, Henan; S7, Jiangsu; S8, Anhui; S9, Zhejiang; S10, Sichuan; S11, Yunnan; S12, Guangxi; S13, Guangdong.

The purity of all standards was ≥ 98%. Chlorogenic acid, cryptochlorogenic acid, rutin, hyperoside, isoquercitrin, astragalin, quercitrin, morusin, mulberroside A, resveratrol, scopoletin, 1-DNJ, and palmitic acid (PA) were purchased from Sigma Aldrich (Darmstadt, Germany). Methanol, acetonitrile (ACN), and formic acid were of chromatography grade (Thermo Fisher Scientific, Waltham, MA, USA). Ultra-pure water was obtained from a Milli-Q water purification system (Millipore, Milford, MA, USA). All other reagents were of analytical grade. Dulbecco’s Modified Eagle’s Medium (DMEM), trypsin-ethylene diamine tetraacetic acid (EDTA) solution, fetal bovine serum (FBS), and phosphate-buffered saline (PBS) were purchased from HyClone (Shanghai, China). The Cell Counting Kit-8 was purchased from EnoGene (CCK-8, Xi’an, China), fluorescein isothiocyanate (FITC) Annexin V Apoptosis Detection Kit I was purchased from Wuhan Seville Biological Technology (Wuhan City, Hubei, China), and a reactive oxygen species (ROS) assay kit was purchased from Biosharp (Guangzhou, China).

### Preparation of samples

#### Preparation of extracts

The fresh mulberry leaf and the mulberry branch were cleaned. The mulberry leaf was dried until the moisture content was less than 10% (at 30°C and 30% humidity). The mulberry branch was cut into thick slices (0.2–0.5 cm in diameter) and dried until the moisture content was less than 10% (at 30°C and 30% humidity). The dried mulberry leaf was crushed and passed through a No 3 sieve (pore size 355 ± 13 μm). Subsequently, 300 g of mulberry branch powder from each planting area and 300 g of mulberry leaf powder were soaked in 2.4 L of 50% ethanol overnight and then subjected to reflux extraction for 3 h, after which the liquid part was collected by filtration. The liquid was concentrated to 3 g/mL (i.e., 1 mL of concentrated extract was equivalent to 3 g of dried mulberry branch sample) under reduced pressure and stored at –20°C.

#### Preparation of test solution

Methanol was added to 0.1 mL of each extract to yield a volume of 10 mL. The suspensions were filtered through a 0.22 μm Millipore filter and used for subsequent composition analysis.

### UPLC chromatographic conditions

An ultra-performance liquid chromatography (UPLC) system fitted with an LC-30AD binary pump (Shimadzu Corporation, Kyoto, Japan), an on-line vacuum degasser, an autosampler, and a column oven were used for similarity analyses. A Poroshell 120 EC-C_18_ column (4.6 mm × 100 mm; 2.7 μm; Agilent Technologies, Santa Clara, CA, USA) was fitted with an EC-C_18_ pre-column (4.6 mm × 5 mm; 2.7 μm; Agilent Technologies, Santa Clara, CA, USA). The column temperature was stabilized to 26°C and the samples were monitored at a wavelength of 320 nm. The mobile phase consisted of ACN (A) and 0.1% (v/v; volume/volume) phosphoric acid in water (B). The flow rate was 0.80 mL/min and the injection volume was 20 μL. The gradient elution program was as follows: 0–3 min, 5%–10% A; 3–30 min, 10%–15% A; 30–40 min, 15%–20% A; 40–75 min, 20%–30% A; 75–80 min, 30%–40% A; 80–100 min, 40%–55% A; 100–110 min, 55%–80% A; 110–115 min, 80%–95% A; then 5% ACN hold for 10 min (He et al., 2020; Polumackanycz et al., 2021; Shreelakshmi et al., 2021).

### Analysis method of UPLC-MS/MS

A UPLC system fitted with an LC-30AD binary pump (Shimadzu Corporation, Japan), an on-line vacuum degasser, an autosampler, and a column oven was used for determining the levels of constituents. A Poroshell 120 EC-C_18_ column (4.6 × 100 mm, 2.7 μm; Agilent, USA) was fitted with an EC-C_18_ pre-column (4.6 × 5 mm, 2.7 μm; Agilent) and the column temperature was stabilized to 26°C. The mobile phase consisted of ACN (A) and 0.1% formic acid (B), with a flow rate of 0.4 mL/min. The injection volume was 5 μL. The gradient elution program was set as follows: 0–5 min, 5%–25% A; 5–20 min, 25% A; 20–34 min, 25%–95% A; then 5% ACN hold for 4 min. The UPLC system was equipped with an API 4000 tandem mass spectrometer (Applied Biosystems/MDS SCIEX, USA) and an electrospray ionization (ESI) source. The quantification was performed using the multiple reactions monitoring (MRM) method. The ESI voltage of positive ions was set to 5,500 V and the ESI voltage of negative ions was set to –4,500 V. Chlorogenic acid, cryptochlorogenic acid, rutin, hyperoside, isoquercitrin, astragalin, quercitrin, morusin, mulberroside A, and resveratrol were detected in the negative ion mode. Scopoletin and 1-DNJ were detected in the positive ion mode (Hu et al., 2017; Ju et al., 2018; D'Urso et al., 2019; Negro et al., 2019; Kim et al., 2020). The mass spectrometry parameters are shown in supplementary **Table S1**.

### Evaluation of similarity between the mulberry branch and leaf extracts

Using the operating conditions described in *UPLC chromatographic conditions*, the similarity between the test solutions described in *Preparation of test solution* was evaluated. The Similarity Evaluation System for Chromatographic Fingerprint Profiles of Chinese Medicines (2012.130723 edition, Chinese Pharmacopoeia Commission) was used to evaluate the similarity of UPLC fingerprints.

### Determination of 12 constituents of mulberry branch and leaf extracts

Using the detection method described in *Analysis method of UPLC-MS/MS*, the level of each constituent in the test solutions described in *Preparation of test solution* was determined. Data acquisition and processing were performed using the Analyst 1.6.2 software [Applied Biosystems (AB Sciex)]. All results were presented as mean ± standard deviation (SD).

### Planting conditions of different planting areas

For each planting area, the following information was obtained through the China Meteorological Data Sharing Service System (http://cdc.nmic.cn/home.do) (**Table 1**): the latitude and longitude, the altitude (m), the annual temperature difference (°C; i.e., the average value of the differences between the highest and lowest temperatures in each of the past 3 years in the planting area), total annual precipitation (mm), rain concentrated (month), the temperature difference in the month before harvest (°C; i.e., the difference between the highest and lowest temperature in the month before harvest), and the precipitation in the month before harvest (mm). The effects of the planting conditions in different planting areas on the constituents of mulberry branches were compared.

### Cell culture and establishment of *in vitro* model

Rat brain microvascular endothelial cells (RBMECs) were obtained from BNCC (Jiangsu, China), and a cell model of hyperglycemia and hyperlipidemia was established with PA and high concentrations of glucose (Bai et al., 2021; Tyagi et al., 2021). RBMECs were cultivated with DMEM containing 10% FBS, 100 U/mL streptomycin, and 100 U/mL penicillin cultured in an incubator at 37°C and 5% CO_2_. The medium was replaced every 2 days and the cells were allowed to adhere to and grow in the culture, covering more than 80% of the bottle. Sterile glucose powder was added to DMEM to prepare a high-sugar medium with a final concentration of 33 mmol/L. A medium containing 200 μmol/L PA hyperglycemia and hyperlipidemia was prepared from a 33 mmol/L high-sugar medium. The cells were cultured in the hyperglycemia and hyperlipidemia medium at 37°C and 5% CO_2_ for 24 h to obtain an *in vitro* hyperglycemia and hyperlipidemia model.

### Cell viability assay

The cell viability was measured using the Cell Counting Kit-8 (CCK-8) test to determine the experimental concentration of mulberry branch extracts from different areas (Pan et al., 2019). The cells were inoculated in a 96-well plate, and the number of cells per well was 5 × 10^3^. The cells were cultured in DMEM containing 10% FBS at 37°C and 5% CO_2_. After adhering to the wall, the cells were divided into a blank control group (no cells), a normal control group, and 12 extract groups; each group comprised six replicate wells. After discarding the medium in the well, 100 μL of serum-free DMEM was added to each well of the blank control group and normal control group, and 100 μL of DMEM containing different concentrations of mulberry branch extract (concentrations of 10^–7^, 10^–6^, 10^–5^, 10^–4^, and 10^–3^ mg/mL of extract were prepared with serum-free DMEM) was added to the extract groups and incubated for 24 h. The original medium was aspirated, followed by the addition of 100 μL serum-free medium and 10 μL of CCK-8 reagent to each well. After culturing for 2 h in the incubator, a microplate reader (Infinite M200 Pro, TECAN, Switzerland) was used to measure the absorbance at 450 nm. The cell viability of different concentrations of mulberry branch extracts was expressed as:

(the absorbance of extract group – the absorbance of blank control group) × 100%/(the absorbance of normal control group – the absorbance of blank control group).

### Apoptosis assay and determination of ROS level

Flow cytometry was performed on a NovoCyte 2040R (ACEA, USA). Apoptosis was detected *via* annexin-V/propidium iodide (PI) double staining (Ma et al., 2018). The cells were inoculated in six-well plates, and the number of cells per well was 1 ×10^6^. The cells were cultured in DMEM containing 10% FBS at 37°C and 5% CO_2_. After adhering to the wall, the cells were divided into the normal control group, hyperglycemia and hyperlipidemia model group, and extract groups, each with six replicate wells. After discarding the medium in the well, 1 mL of medium was added to each well of the normal control group, 1 mL of hyperglycemia and hyperlipidemia medium to each well of the hyperglycemia and hyperlipidemia model group, and 1 mL of medium with an extract concentration of 10^–4^ mg/mL prepared with hyperglycemia and hyperlipidemia medium to each well of the extract groups. The cells were cultured in an incubator for 24 h. The cells in each group were subsequently washed once with PBS and then digested with trypsin in the absence of Ethylene Diamine Tetraacetic Acid (EDTA), followed by washing twice with PBS. Thereafter, 200 μL of buffer was added to the harvested cells. Next, 5 μL of annexin-V-fluorescein isothiocyanate (FITC) was added to the cells and they were kept in the dark for 15 min at room temperature, followed by the addition of 5 μL of PI staining solution and incubation for another 5 min. Apoptosis was detected using flow cytometry. The NovoExpress software version 1.2.1 (ACEA Biosciences Inc.) was used to generate scatter plots. The number of annexin-V-fluorescein isothiocyanate (FITC)- and PI-positive cells was used to calculate the cellular apoptotic rate.

Reactive oxygen detection was performed using the fluorescent probe, 2',7'-Dichlorodihydrofluorescein diacetate (DCFH-DA), which reacted with intracellular ROS, generating fluorescent products (Liu et al., 2021b). The cells were inoculated in a 12-well plate with round coverslips, and the number of cells per well was 1 × 10^5^. The cells were cultured in DMEM containing 10% FBS at 37°C and 5% CO_2_. After adhering to the wall, the cells were divided into the normal control group, hyperglycemia and hyperlipidemia model group, and extract groups, each with six replicate wells. After discarding the medium in the well, 1 mL of medium was added to each well of the normal control group, 1 mL of hyperglycemia and hyperlipidemia medium to each well of the hyperglycemia and hyperlipidemia model group, and 1 mL of medium with an extract concentration of 10^–4^ mg/mL prepared with hyperglycemia and hyperlipidemia medium to each well of the extract groups. The cells were then cultured in an incubator in the dark for 24 h and then washed with a serum-free medium, followed by incubation with 10 μM DCFH-DA in a cell incubator (maintained at 37°C) in the dark for 30 min. Subsequently, the cells were washed twice with PBS to fully remove the DCFH-DA that did not enter the cells. The fluorescence intensity of the cells was detected using an inverted fluorescence microscope (Olympus IX53+DP73, Japan) at an excitation wavelength (λ_ex_) of 488 nm and an emission wavelength (λ_em_) of 525 nm. Image J software was used to detect the fluorescence intensity of the cells in the captured cell images. One-way analysis of variance (ANOVA) in GraphPad Prism 8 software (San Diego, CA, USA) was used to analyze the data. Tukey’s test was used for comparison between groups. A *p*-value < 0.05 was considered statistically significant.

### Correlation analysis of the influence of planting conditions on constituents and bioactivities

PLSR analysis was performed on the planting conditions described in *Planting conditions of different planting areas* and constituent levels described in *Determination of 12 constituents of mulberry branch and leaf extracts* to reveal the effects of planting conditions on constituent similarities and levels. MFA was performed on the constituent levels described in *Determination of 12 constituents of mulberry branch and leaf extracts* and the bioactivities described in *Apoptosis assay and determination of ROS level* to reveal the relationship between constituent levels and bioactivities. ROS can cause oxidative damage; therefore, antioxidation activity can be assessed by ROS-scavenging rate. The calculation methods for antiapoptotic and ROS-scavenging rates were as follows:

Antiapoptotic = (the mean value of apoptosis rate in the model group – the mean of the apoptosis rate in the extract group)/(the mean value of apoptosis rate in the model group – the mean of the apoptosis rate in the normal group).

ROS-scavenging rate = (the mean value of fluorescence intensity in the model group – the mean value of fluorescence intensity in the extract group) × 100%/(the mean value of fluorescence intensity in the model group – the mean value of fluorescence intensity in the normal group).

## Results and discussion

### The influence of planting conditions on the similarities and levels of constituents of mulberry branches

The mulberry tree has low survival requirements. If sufficient light is available, it is highly adaptable to differences in temperature and soil pH, which allows for successful planting in different geographical regions. Studies have shown that the secondary metabolism of mulberry trees can undergo huge variations (Sánchez-Salcedo et al., 2016; Yang et al., 2017). These secondary metabolites are the main constituents of mulberry trees that produce bioactivities. Changes in planting conditions may be an important cause of differences in levels of constituents. Although mulberry is classified as a thermophilic plant, it can withstand temperatures of –30°C and its stem can regenerate rapidly after freezing (Blitek et al., 2022). Therefore, it is necessary to study the changes in the levels of the constituents of the mulberry branch in combination with planting conditions.

**Figure 1:**
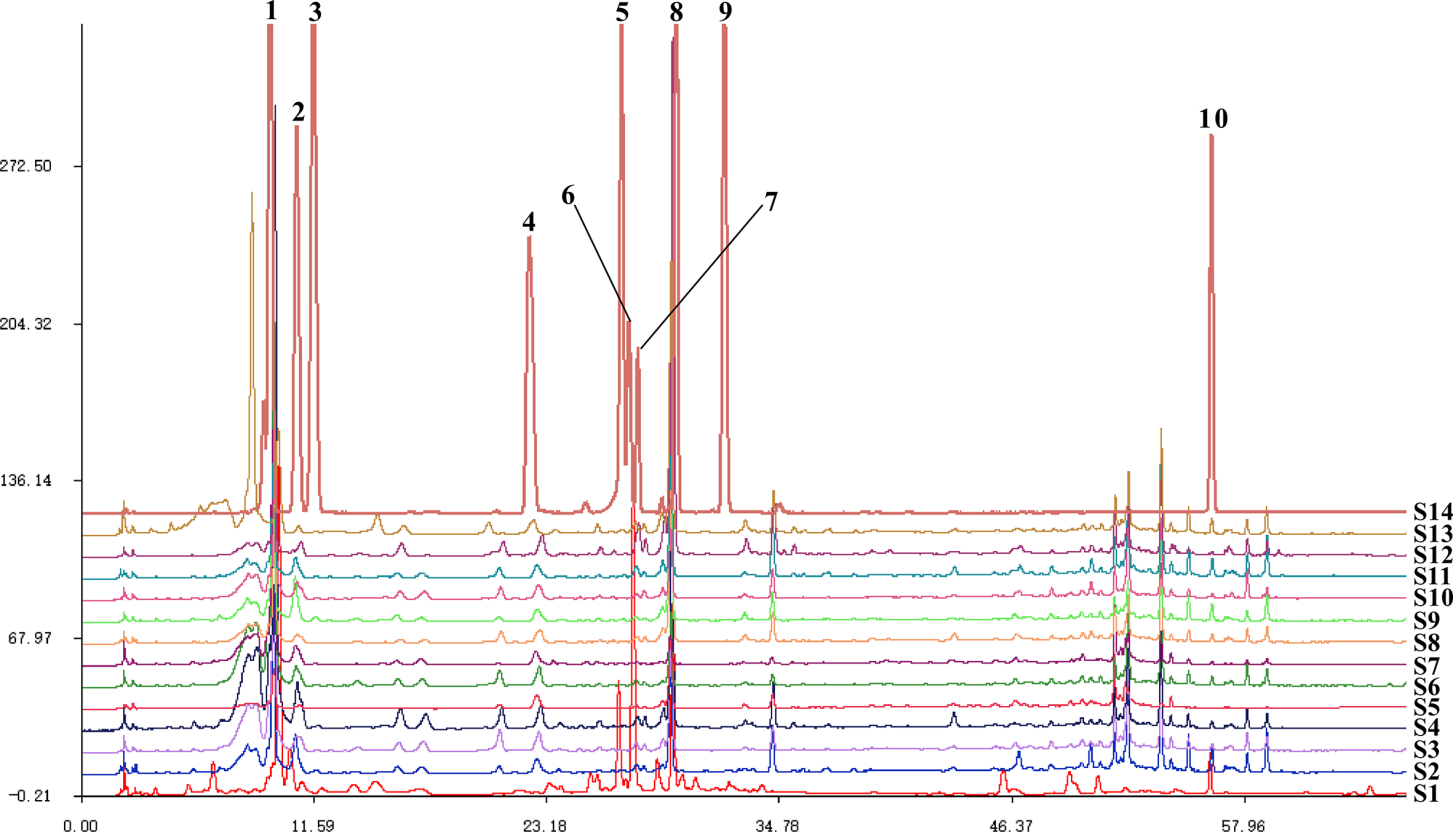
The fingerprints of mulberry branch extracts from 12 planting areas. S1, Mulberry leaf; S2, Heilongjiang; S3, Liaoning; S4, Hebei; S5, Shanxi; S6, Henan; S7, Jiangsu; S8, Anhui; S9, Zhejiang; S10, Sichuan; S11, Yunnan; S12, Guangxi; S13, Guangdong; S14, Mixed standard. 1, mulberroside A; 2, chlorogenic acid; 3, cryptochlorogenic acid; 4, scopoletin; 5, rutin; 6, isoquercitrin and hyperoside; 7, astragalin; 8, quercitrin; 9, resveratrol; 10, morusin.

The Similarity Evaluation System for Chromatographic Fingerprint Profiles of Chinese Medicines was used to obtain the similarity matrix of the mulberry branch from 12 planting areas (**Table 2** and **Figure 1**). The planting conditions at different planting areas are listed in **Table 1** (data obtained from public information on the internet and research reports). From the comparison of the numbers and retention times of chromatographic peaks, the same constituents of the mulberry leaf and the mulberry branch were found mainly in peaks 1–9, including mulberroside A, chlorogenic acid, cryptochlorogenic acid, scopoletin, rutin, isoquercitrin, hyperoside, astragalin, quercitrin, and resveratrol. The unique compound contained in the mulberry branch was peak 10, which was morusin. The above results indicate significant similarity between the mulberry leaf and branch except for branches from a few producing regions, such as Guangxi and Guangdong, which showed less similarity. The author used the established UPLC-mass spectra (MS)/MS method to determine the levels of 12 constituents of the mulberry branch and leaf (**Table 3**). The results revealed that the levels of morusin, mulberroside A, and 1-DNJ in the mulberry branch from almost all producing areas were higher than in the mulberry leaf, while other constituents, such as chlorogenic acid, cryptochlorogenic acid, rutin, hyperoside, isoquercitrin, astragalin, and quercitrin, were lower than in the mulberry leaf. The levels of chlorogenic acid, cryptochlorogenic acid, and isoquercitrin were the highest in S11; the levels of rutin, hyperoside, and morusin were the highest in S13; the levels of astragalin, mulberroside A, resveratrol, and 1-DNJ were the highest in S4; and the levels of quercitrin and scopoletin were the highest in S7.

**Table 2:** Similarity of mulberry branches from different planting areas.

	S1	S2	S3	S4	S5	S6	S7	S8	S9	S10	S11	S12	S13	Reference fingerprint
S1	1	0.185	0.154	0.153	0.262	0.111	0.094	0.275	0.267	0.153	0.245	0.338	0.367	0.38
S2	0.185	1	0.922	0.904	0.672	0.897	0.87	0.864	0.92	0.958	0.955	0.529	0.269	0.93
S3	0.154	0.922	1	0.98	0.614	0.987	0.94	0.843	0.856	0.966	0.835	0.444	0.174	0.922
S4	0.153	0.904	0.98	1	0.608	0.976	0.938	0.848	0.873	0.951	0.8	0.468	0.168	0.921
S5	0.262	0.672	0.614	0.608	1	0.519	0.556	0.834	0.794	0.632	0.768	0.789	0.395	0.764
S6	0.111	0.897	0.987	0.976	0.519	1	0.949	0.771	0.806	0.95	0.773	0.329	0.109	0.875
S7	0.094	0.87	0.94	0.938	0.556	0.949	1	0.727	0.781	0.907	0.743	0.289	0.205	0.855
S8	0.275	0.864	0.843	0.848	0.834	0.771	0.727	1	0.954	0.854	0.902	0.841	0.384	0.945
S9	0.267	0.92	0.856	0.873	0.794	0.806	0.781	0.954	1	0.868	0.922	0.756	0.355	0.95
S10	0.153	0.958	0.966	0.951	0.632	0.95	0.907	0.854	0.868	1	0.892	0.471	0.207	0.924
S11	0.245	0.955	0.835	0.8	0.768	0.773	0.743	0.902	0.922	0.892	1	0.682	0.368	0.915
S12	0.338	0.529	0.444	0.468	0.789	0.329	0.289	0.841	0.756	0.471	0.682	1	0.481	0.687
S13	0.367	0.269	0.174	0.168	0.395	0.109	0.205	0.384	0.355	0.207	0.368	0.481	1	0.449
Reference fingerprint	0.38	0.93	0.922	0.921	0.764	0.875	0.855	0.945	0.95	0.924	0.915	0.687	0.449	1

S1, Mulberry leaf; S2, Hei long jiang; S3, Liao ning; S4, He bei; S5, Shan xi; S6, He nan; S7, Jiang su; S8, An hui; S9, Zhe jiang; S10, Si chuan; S11, Yun nan; S12, Guang xi; S13, Guang dong.

**Table 3:** Levels of 12 constituents of extracts (*n* = 3).

Constituent	Level (μg/g)												
	S1	S2	S3	S4	S5	S6	S7	S8	S9	S10	S11	S12	S13
Chlorogenic acid	432.68 ± 29.74^a^	152.59 ± 6.91^e^	65.63 ± 4.31^f^	335.84 ± 13.2^c^	14 ± 0.68^h^	87.35 ± 3.85^f^	179.59 ± 9.58^e^	59.38 ± 3.6^f,g^	264.47 ± 1.72^d^	152.97 ± 3.67^e^	389.08 ± 25.2^b^	25.69 ± 1.29^g,h^	149.11 ± 8.12^e^
Cryptochlorogenic acid	82.97 ± 6.6^a^	12.48 ± 0.35^e,f,g^	8.97 ± 0.57^f,h^	41.28 ± 0.48^c^	2.12 ± 0.11^h^	11.37 ± 0.87^f,g^	17.01 ± 0.97^e^	7.24 ± 0.33^f,g,h^	30.99 ± 2.24^d^	22.28 ± 0.95^e^	63.97 ± 5.26^b^	4.64 ± 0.23^f,g,h^	15.29 ± 0.96^e,f^
Rutin	47.5 ± 2.53^a^	3.22 ± 0.12^b,c^	1.57 ± 0.06^b,c,d,e^	2.04 ± 0.12^b,c,d,e^	0.43 ± 0.01^e^	1.82 ± 0.07^b,c,d,e^	1.03 ± 0.04^d,e^	1.4 ± 0.09^c,d,e^	2.42 ± 0.21^b,c,d,e^	1.26 ± 0.1^c,d,e^	2.83 ± 0.14^b,c,d^	0.84 ± 0.05^d,e^	3.65 ± 0.3^b^
Hyperoside	59.43 ± 0.67^a^	5.11 ± 0.04^c^	1.91 ± 0.12^f^	4.51 ± 0.27^c,d^	0.64 ± 0.04^h^	1.91 ± 0.07^f^	0.88 ± 0.01^g,h^	1.47 ± 0.11^f,g^	1.83 ± 0.06^f^	2.07 ± 0.14^e,f^	4.38 ± 0.38^d^	2.76 ± 0.23^e^	6.29 ± 0.01^b^
Isoquercitrin	66.27 ± 4.72^a^	5.85 ± 0.45^d^	2.68 ± 0.05^g^	9.45 ± 0.32^b^	0.95 ± 0.03^g^	2.32 ± 0.14^g^	4.58 ± 0.18^e^	2.18 ± 0.13^g^	3.77 ± 0.2^f^	3.67 ± 0.3^f,g^	8.13 ± 0.35^b^	3.39 ± 0.09^g^	6.44 ± 0.33^c^
Astragalin	63.5 ± 5.39^a^	0.41 ± 0.01^b^	0.17 ± 0.01^b^	1.6 ± 0.13^b^	0.23 ± 0.02^b^	0.14 ± 0^b^	0.61 ± 0.01^b^	0.39 ± 0.01^b^	0.46 ± 0.02^b^	0.47 ± 0.02^b^	0.66 ± 0.03^b^	1.04 ± 0.04^b^	1.2 ± 0.03^b^
Quercitrin	0.27 ± 0.02^a^	0.01 ± 0^d^	0.01 ± 0^d^	0.01 ± 0^d^	0.01 ± 0^d^	0.01 ± 0^d^	0.04 ± 0^b^	0.04 ± 0^b^	0.02 ± 0^c,d^	0.01 ± 0^d^	0.01 ± 0^d^	0.03 ± 0.01^b,c^	0.01 ± 0^d^
Morusin	—	251.04 ± 13.04^d,e,f^	248.44 ± 16.07^d,e,f^	298.97 ± 21.51^b,c^	27.79 ± 1.23^h^	277.79 ± 14.06^b,d^	145.53 ± 10.36^g^	273.71 ± 15.85^b,c,d^	212.03 ± 11.28^f^	357.67 ± 18.48^a^	276.68 ± 6.14^b,e^	260.7 ± 7.72^c,d,e^	308.63 ± 20.95^b^
Mulberroside A	11.71 ± 0.38^f^	—	—	2271.32 ± 174.73^a^	73.44 ± 3.59^f^	362.6 ± 28.18^d^	1157.57 ± 66.61^b^	241.76 ± 3.74^d,e^	262.98 ± 9.24^d,e^	376.63 ± 15.02^d^	697.21 ± 62.3^c^	—	194.88 ± 10.6^e,f^
Resveratrol	0.88 ± 0.06^f,g^	0.49 ± 0.03^g^	0.64 ± 0.03^g^	5.69 ± 0.4^a^	1.29 ± 0.07^e,f^	0.4 ± 0.03^g^	1.85 ± 0.1^c,d^	1.85 ± 0.08^c,d^	1.91 ± 0.13^c^	1.39 ± 0.12^c,e^	4.6 ± 0.36^b^	1.83 ± 0.09^c^	1.81 ± 0.09^c^
Scopoletin	10.11 ± 0.7^c^	2.93 ± 0.25^d^	8.32 ± 0.2^c^	8.63 ± 0.77^c^	9.53 ± 0.61^c^	8.76 ± 0.44^c^	22.06 ± 1.29^a^	10.37 ± 0.91^c^	20.63 ± 1.78^a^	17.38 ± 1.29^b^	10.59 ± 0.76^c^	17.6 ± 0.61^b^	10.31 ± 0.64^c^
1-DNJ	201.56 ± 4.67^h^	606.63 ± 12.87^g^	1085.06 ± 32.01^b^	2102.19 ± 89.56^a^	219.01 ± 4.51^h^	934.37 ± 53.97^c,d^	580.06 ± 28.02^g^	642.91 ± 50.59^f,g^	757.91 ± 36.11^e,f^	995.83 ± 20.62^b,c^	1031.52 ± 47.04^b,c^	648.86 ± 43.02^f,g^	847.13 ± 47.21^d,e^

1-DNJ, 1-deoxynojirimycin; S1, Mulberry leaf; S2, Hei long jiang; S3, Liao ning; S4, He bei; S5, Shan xi; S6, He nan; S7, Jiang su; S8, An hui; S9, Zhe jiang; S10, Si chuan; S11, Yun nan; S12, Guang xi; S13, Guang dong. The same letter in the two groups of data indicates no significant difference. Different letters in the two groups of data indicate a significant difference (p < 0.05). The letters a-h represent the results of the analysis of variance. In the two groups of data, the same letter represents no significant difference, but different letters represent significant difference.

### Mulberry branch extract can reduce the apoptosis rate and inhibit oxidative damage of RBMEC in a hyperglycemia and hyperlipidemia environment

**Figure 2:**
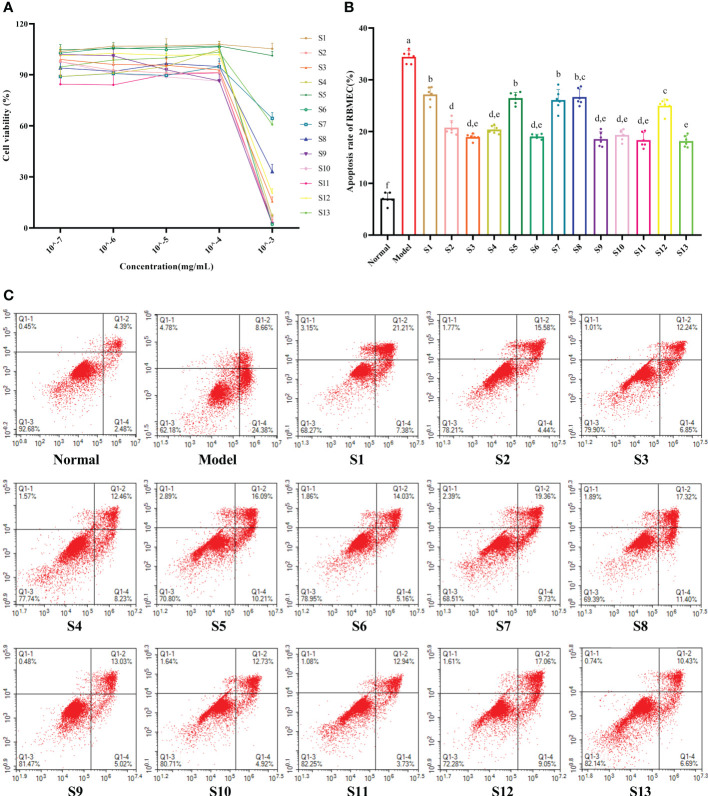
Mulberry branch can reduce the apoptosis of rat brain microvascular endothelial cells (RBMECs) in hyperglycemia and hyperlipidemia. S1, Mulberry leaf; S2, Heilongjiang; S3, Liaoning; S4, Hebei; S5, Shanxi; S6, Henan; S7, Jiangsu; S8, Anhui; S9, Zhejiang; S10, Sichuan; S11, Yunnan; S12, Guangxi; S13, Guangdong. **(A)**, CCK-8 assay to determine the effect of different concentrations mulberry branch extracts on the viability of cells; **(B)**, Quantitative analysis of the apoptosis rates of RBMECs treated with different mulberry branch extracts for 24 h; **(C)**, annexin-V/propidium iodide (PI) double staining followed by cytometry analysis was performed to evaluate the cell apoptosis of RBMEC treatment with different mulberry branch extracts for 24 h. The same letter in the two groups of data indicates no significant difference. Different letters in the two groups of data indicate a significant difference (*p* < 0.05).

As shown in **Figure 2A**, when the extract concentration in the medium was 10^–7^–10^–4^ mg/mL, there was no significant change in the cell viability of the RBMEC (*p* > 0.05). Therefore, 10^–4^ mg/mL was chosen as the experimental concentration *in vitro*. The RBMECs were double stained by annexin-V-fluorescein isothiocyanate (FITC)/PI and then subjected to flow cytometry to test the protective effects of the mulberry branch extracts against the damage induced by the hyperglycemia and hyperlipidemia media. The experimental results showed that the cellular apoptotic rate of the model group (34.43%) was significantly higher than that of the normal control group (7.07%, *p* < 0.05). After treatment with mulberry branch extracts, apoptotic rates were significantly decreased (*p* < 0.05, relative to model group) (**Figures 2B, C**). Among them, the antiapoptotic effects of the S2, S3, S4, S6, S9, S10, S11, S12, and S13 groups were significantly lower than that of the mulberry leaf group (*p* < 0.05).

**Figure 3:**
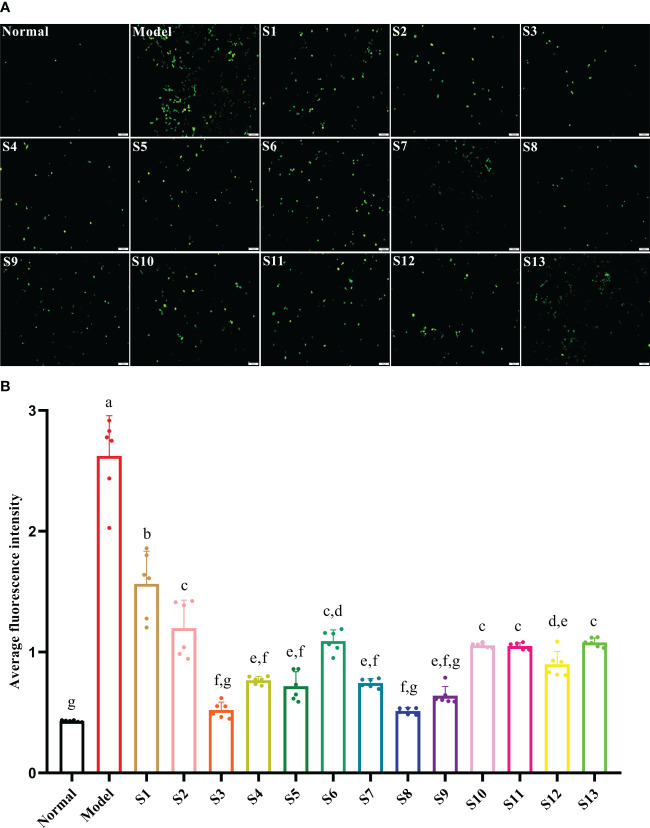
Mulberry branch can reduce the apoptosis of rat brain microvascular endothelial cells (RBMECs) in hyperglycemia and hyperlipidemia. S1, Mulberry leaf; S2, Heilongjiang; S3, Liaoning; S4, Hebei; S5, Shanxi; S6, Henan; S7, Jiangsu; S8, Anhui; S9, Zhejiang; S10, Sichuan; S11, Yunnan; S12, Guangxi; S13, Guangdong. **(A)**, Fluorescence probe DCFH-DA was used to detect the level of reactive oxygen species (ROS) generated owing to hyperglycemia and hyperlipidemia in an *in vitro* model after treatment with different branch extracts for 24 h; **(B)**, Quantitative analysis of cell fluorescence intensity of different mulberry branch extracts treated for 24 h. The same letter in the two groups of data indicates no significant difference. Different letters in the two groups of data indicate a significant difference (*p* < 0.05).

The intracellular ROS in response to the hyperglycemia and hyperlipidemia environment was significantly increased to 510% that of the normal control group (*p* < 0.05), which eventually led to cell oxidative damage. Compared with the model, all mulberry branch extracts (10^–4^ mg/mL) had the ability to inhibit the intracellular production of ROS and reduce oxidative damage in the RBMECs after exposure to hyperglycemia and hyperlipidemia media, to varying degrees, and the effect was significantly stronger than in the mulberry leaf group (**Figures 3A, B**, *p* < 0.05). Among them, S3 and S8 had the most significant effects.

ROS is an oxidative factor that is closely related to the oxidative stress response of cells, and a hyperglycemia and hyperlipidemia environment can induce vascular endothelial cells to produce more ROS, causing oxidative damage to cells and destroying capillary structures (Rendra et al., 2019; Cao et al., 2021). As the damage intensifies, it will eventually lead to the apoptosis of vascular endothelial cells and cause microcirculatory complications in multiple chronic diseases (Chalimeswamy et al., 2021). Bai et al. found that mulberry extract had a protective effect on the increase of ROS and vascular endothelial damage caused by high sugar and high fat (Bai et al., 2021). Ranjan et al. found that mulberry extract could achieve its antioxidant effect by reducing the activities of catalase, serum glutamic oxaloacetic transaminase, and serum glutamic pyruvic transaminase in mice used as diabetes models (Ranjan et al., 2017). This research found that the mulberry branch and mulberry leaf had highly similar bioactivities, and the antioxidant and antiapoptotic effects of the mulberry branch were even greater than the mulberry leaf (**Figures 2B** and **3B**). These results provide support for the value of developing the mulberry branch as a new economic plant resource.

### Correlation analysis of planting conditions and bioactivities

**Figure 4:**
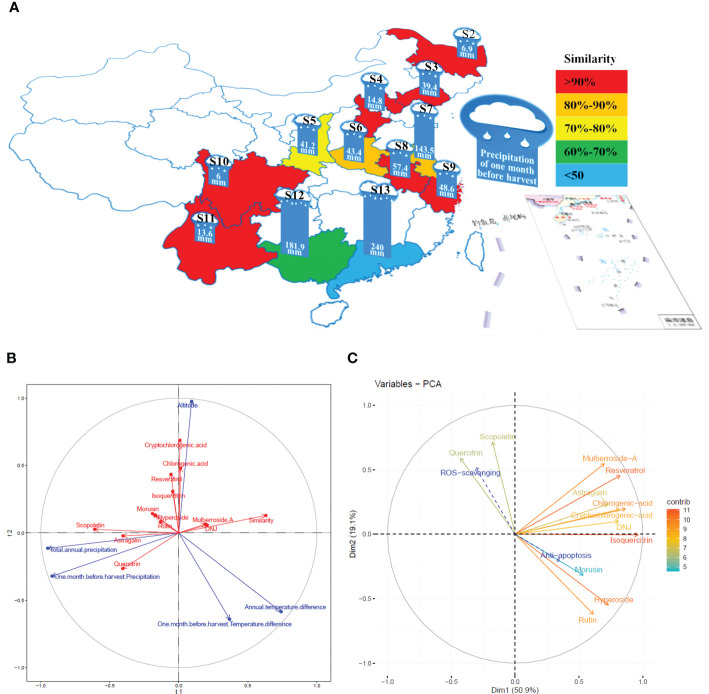
The correlation between the planting conditions of the planting areas, the constituents of the mulberry branch extract and the bioactivity variations were analyzed by partial least square regression (PLSR) and the “FactoMineR” software. S2, Heilongjiang; S3, Liaoning; S4, Hebei; S5, Shanxi; S6, Henan; S7, Jiangsu; S8, Anhui; S9, Zhejiang; S10, Sichuan; S11, Yunnan; S12, Guangxi; S13, Guangdong. **(A)** Precipitation in the month before harvest and the similarity of mulberry branches from different planting areas. Similarity: red > 0.9 (S2, S3, S4, S8, S9, S10, S11); 0.8 < orange < 0.9 (S6, S7); 0.7 < yellow < 0.8 (S5); 0.6 < green < 0.7 (S12); blue < 0.6 (S13). **(B)** The effects of planting conditions on the levels of constituents and the similarity of mulberry branches analyzed by PLSR: blue (planting conditions), red (constituents). The vector length of each composition represents the amount of change. The greater the change, the longer the vector length. The angle between the vector direction of the composition and the planting conditions represents the strength of the correlation. The more similar, the stronger the correlation is, and opposite direction represents negative correlation. **(C)** multiple factor analysis (MFA) based on reactive oxygen species (ROS) and apoptosis results in line with the constituents of mulberry branch extracts: the vector length and color of each composition in the mulberry branch extracts represent the contribution of the composition to the overall efficacy. Color of vector (contribution degree, red direction represents greater contribution value, while blue direction represents the opposite), the angle between the vector direction of composition contribution (solid line arrow), and variation in specific activity (dotted line arrow) represents the strength of the correlation: a smaller angle indicates stronger correlation.

PLSR was used to determine the influence of altitude, annual temperature difference, total annual precipitation, temperature differences in the month before harvest, and precipitation in the month before harvest on the levels and similarities of 12 constituents of the mulberry branch (**Figures 4A, B**). The results indicated that the directions of the three factors of altitude, precipitation, and temperature were broadly symmetrical but distinguishable. Among the 12 studied constituents, chlorogenic acid, cryptochlorogenic acid, isoquercitrin, and resveratrol showed a similar direction to altitude, suggesting a positive correlation. These constituents also had a weakly negative correlation with temperature difference. Therefore, the levels of these constituents were most significantly affected by altitude, indicating that mulberry branches harvested from higher altitudes tended to be rich in these constituents. In contrast, a large temperature difference tended to reduce the amount of these constituents. For example, the altitudes of S3 and S9 were almost the same, but because the temperature difference of S9 was significantly lower than that of S3, the levels of chlorogenic acid, cryptochlorogenic acid, isoquercitrin, and resveratrol were higher in S9 than in S3. This finding was consistent with the previous study by Blitek et al., in which they also showed that the levels of chlorogenic acid, cryptochlorogenic acid, isoquercitrin, and resveratrol were influenced by temperature difference and altitude (Blitek et al., 2022). In addition, the directions of these four constituents were almost perpendicular to precipitation, indicating a negligible effect of precipitation on the levels of these constituents. By applying the same analysis method, rutin, hyperoside, and morusin were found to have a strong negative correlation with temperature difference and a weak positive correlation with altitude and precipitation, revealing that temperature difference had a greater impact, and the levels of these three constituents were higher in areas with small temperature differences. For instance, S10, S11, and S13 varied greatly in altitude but had small temperature differences, and the levels of rutin, hyperoside, and morusin were similarly high among all three. Precipitation was negatively correlated with mulberroside A and 1-DNJ but had a strong positive correlation with astragalin, quercitrin, and scopoletin. The precipitation in the month before harvest had an average effect on astragalin and quercitrin, while astragalin was most significantly affected by total annual precipitation. For example, the annual precipitation in S2 and S4 was similar, but owing to more precipitation in the month before harvest in S4, the levels of astragalin and quercitrin were higher in S4 than in S2. In general, precipitation was negatively correlated with the similarity of mulberry branches in different planting areas, suggesting that it was an important influencing factor. In contrast, the altitude and temperature difference were almost vertical to the similarity, suggesting that these factors had little effect on similarity. Therefore, it is inferred that precipitation may be an important climatic factor affecting the similarities in the composition of mulberry branches.

MFA was conducted to analyze the correlation between the levels of 12 constituents and the variations of two bioactivities in mulberry branch extracts from different planting areas (**Figure 4C**). The results showed that the 12 constituents contributed to the antiapoptotic and antioxidant activities of mulberry branches to varying degrees. Mulberroside A, chlorogenic acid, cryptochlorogenic acid, astragalin, resveratrol, isoquercitrin, and 1-DNJ had similar contributions to these two bioactivities of the mulberry branch. The sum of the contributions of these seven constituents accounted for more than half of the total efficacy. Their directions were all in the first quadrant, which was broadly perpendicular to the direction of antiapoptosis and antioxidative effect, indicating that these constituents were, in general, not related to the variation among mulberry branch extracts from different planting areas. In other words, these constituents together produced the main bioactivities but had little effect on the magnitude of bioactive differences of mulberry branch extracts from different planting areas. For example, the levels of mulberroside A, chlorogenic acid, cryptochlorogenic acid, astragalin, resveratrol, and 1-DNJ in S10 and S11 were very distinctive, but their antiapoptosis and antioxidative effects were not significantly different.

Previous studies have reported on a range of bioactivities and the constituents of mulberry. Chlorogenic acid showed strong antioxidant activity at 200 mg/kg (Metwally et al., 2020). Mulberroside A in the water extract of Cortex Mori also had good antioxidant activity at 0.58 g/mL (Xu et al., 2021). A basic diet containing 500 mg/kg resveratrol could reverse the oxidative damage of hepatocytes (Liu et al., 2021a), and 5 mg/L of 1-DNJ could enhance the antioxidant activity of tilapia splenocytes (Tang et al., 2017). Similar activities were also found in cryptochlorogenic acid and isoquercitrin (Zhou, 2020). Rutin could alleviate H9C2 cell damage induced by high glucose levels by inhibiting apoptosis and endoplasmic reticulum stress (Wang et al., 2021). Hyperoside significantly inhibited apoptosis induced by high glucose levels in a dose-dependent manner (Wu et al., 2020). When the concentration of quercitrin reached 160 μg/mL, it could significantly increase the survival rate of human embryonic kidney 293 T cells (Li et al., 2020). Scopoletin prevented palmitic acid and bile acid-induced hepatocyte death by inhibiting endoplasmic reticulum stress and ROS generation and reducing the phosphorylation of JNK, one of the cell death signaling intermediates (Wu et al., 2022). These studies have shown that various constituents of the mulberry branch have clear antiapoptotic and antioxidant effects, which are the basis for the bioactivities of the mulberry branch. Mulberroside A, chlorogenic acid, cryptochlorogenic acid, astragalin, resveratrol, and 1-DNJ in the mulberry branch could act together in a combined form in the extract through various pathways, resulting in stable and basic antiapoptotic and antioxidant relatively. Therefore, it is believed that these constituents constitute the basic antiapoptotic and antioxidant effects of the mulberry branch and are the foundation of the bioactive function of the mulberry branch, but are not the elements that cause the differences in the bioactive effects of mulberry branches from different planting areas.

The analysis results show that the antiapoptotic and antioxidative effects of the mulberry branch are two vectors with opposite directions. Some constituents of the mulberry branch had the same or similar directions as the antiapoptotic or antioxidant effects, indicating that these constituents were major contributors to the variations in antiapoptotic or antioxidant activities observed among mulberry branch extracts from different planting areas. Rutin, hyperoside, and morusin showed the same direction as the antiapoptotic effect but the opposite direction to the antioxidative effect, which means that these constituents were positively correlated with the variations in antiapoptosis activity among mulberry branch extracts but negatively correlated with the change in antioxidative capability. Thus, the results might suggest that these three constituents are beneficial for the antiapoptosis function of the mulberry branch but unfavorable for the antioxidant activity of the mulberry branch. Compared with the above three constituents, scopoletin and quercitrin showed almost the opposite influence on antiapoptotic and antioxidation activities. Owing to this relative affect trend, the ratio of these five constituents is probably the key factor for the differences in the bioactivity of mulberry branches from different planting areas and is perhaps more important than the levels of these constituents. Planting conditions can have a direct impact on the ratio of these key constituents. According to the analysis shown in **Figure 4B**, the annual temperature difference and the temperature difference in the month before harvest had a greater impact on the levels of rutin, hyperoside, and morusin. In areas where both these temperature differences were small, the amounts of rutin, hyperoside, and morusin were relatively high, e.g., in S13. In contrast, the total annual precipitation and precipitation in the month before harvest had a greater impact on the levels of quercitrin and scopoletin thus, quercitrin and scopoletin were relatively rich in areas with higher total annual precipitation and higher precipitation in the month before harvest. For example, S8 had more precipitation than S6 and so the levels of quercitrin and scopoletin in S8 were higher, which in turn affected the bioactivity of the mulberry branch in these two areas. Extracts of S8 had a certain advantage in antioxidation, while S6 was more effective in antiapoptosis. It can be seen that planting conditions have a significant impact on the content and ratio of the constituents of plants (Kreft et al., 2022), which leads to the bioactivities bias in mulberry branches from different planting areas. Thus, clarifying the relationship between planting conditions and constituents has particular significance for the utilization of bioactive resources in the mulberry branch after harvesting.

## Conclusions

In this study, the correlation between planting conditions (such as altitude, temperature difference, and precipitation) in different planting areas and bioactivities in the mulberry branch was determined and analyzed. Specifically, the research found that mulberry the branch has similar constituents and bioactivities to the mulberry leaf. Comprehensive analysis suggested that the main constituents of mulberry branches in different planting areas were similar, but the bioactivities were biased. Among the 12 constituents tested in this study, seven constituents (chlorogenic acid, cryptochlorogenic acid, isoquercitrin, astragalin, mulberroside A, resveratrol, and 1-DNJ), together, probably determined the basal level of the mulberry branch’s bioactivity, which is fundamental to the potential of the mulberry branch’s bioactive resources to make mulberry an economic plant. Mulberry branches from different planting areas exhibited differences in antiapoptosis and antioxidative damage capacities. These differences were mainly caused by five key constituents (rutin, hyperoside, quercitrin, morusin, and scopoletin). Climatic factors, particularly precipitation and temperature difference, in the planting areas significantly affected the levels and ratios of these constituents. The relationship between the planting conditions, constituents, and bioactivities of the mulberry branch was analyzed by PLSR and MFA. The results showed that planting conditions with smaller temperature differences would make the antiapoptotic effect of the mulberry branch more prominent and that adequate irrigation would promote the antioxidative effect of the mulberry branch. These results not only indicate that the mulberry branch has potential use as a new economic plant part but also guide the rational utilization of mulberry branches for different medical needs, thus reducing the waste of resources. The mathematical analysis method used in this study provides data supporting the selection of planting conditions to utilize the bioactivities of mulberry.

## Data availability statement

The original contributions presented in the study are included in the article/**Supplementary Material**. Further inquiries can be directed to the corresponding author.

## Author contributions

HB and SJ conceptualized and wrote the original draft. HB and JL carried out the experimental part under the supervision of YL. YT and XZ critically reviewed the draft. YX conducted the statistical analysis of the experimental data. SW and PJ procured research funding. All authors contributed to the article and approved the submitted version.
