# Supplementary material for: Planting conditions can enhance the bioactivity of mulberry by affecting its composition
Source: Front Plant Sci. 2023 Mar 7;14:1133062. doi: 10.3389/fpls.2023.1133062 (PMC10028076; doi:10.3389/fpls.2023.1133062)
Supplement: Supplementary file 1 [file Table_1.docx]

Supplementary Material

**Effects of planting conditions on bioactivity of mulberry: A mathematical analysis of compositions and function differences**

Huixin Bai^1,a^, Shanfeng Jiang^2,a^, Jincai Liu^1^, Ye Tian^2^, Xiaohui Zheng^1^, Siwang Wang^1^, Yanhua Xie^1^, Yao Li^3*^, Pu Jia^1*^

*** Correspondence:** Corresponding Author: jiapu77@126.com

# Supplementary Table

**Table S1 MS parameters and retention times (RTs) of compositions including declustering potential (DP) and collision energy (CE)**

| **Compositions** | **Precursor ion (m/z)** →**Product ion (m/z)** | **DP(V)** | **CE(V)** | **RT(min)** |
| --- | --- | --- | --- | --- |
| Chlorogenic acid | 353.0→191.0 | -61.0 | -31.0 | 7.11 |
| Cryptochlorogenic acid | 352.9→172.7 | -60.0 | -23.0 | 7.30 |
| Rutin | 609.0→300.8 | -141.0 | -46.0 | 8.78 |
| Hyperoside | 463.0→300.0 | -119.1 | -38.9 | 9.29 |
| Isoquercitrin | 463.0→300.8 | -101.0 | -29.0 | 9.36 |
| Astragalin | 447.0→284.0 | -102.0 | -38.9 | 10.51 |
| Quercitrin | 447.1→300.0 | -103.1 | -36.1 | 10.76 |
| Morusin | 419.0→297.0 | -138.6 | -32.8 | 33.33 |
| Mulberroside A | 567.0→243.0 | -118.1 | -36.1 | 6.41 |
| Resveratrol | 227.0→185.0 | -86.5 | -26.2 | 17.19 |
| Scopoletin | 193.0→133.0 | 88.0 | 27.0 | 10.61 |
| 1-DNJ | 164.0→146.2 | 82.8 | 16.7 | 2.24 |
